# Studying the roles of salt ions in the pore initiation and closure stages in the biomembrane electroporation

**DOI:** 10.1063/5.0147104

**Published:** 2023-05-08

**Authors:** Qiongyao Mou, Mengli Xu, Jinan Deng, Ning Hu, Jun Yang

**Affiliations:** Key Laboratory of Biorheological Science and Technology, Ministry of Education and Bioengineering College, Chongqing University, Chongqing 400044, China

## Abstract

Electroporation shows great potential in biology and biomedical applications. However, there is still a lack of reliable protocol for cell electroporation to achieve a high perforation efficiency due to the unclear influence mechanism of various factors, especially the salt ions in buffer solution. The tiny membrane structure of a cell and the electroporation scale make it difficult to monitor the electroporation process. In this study, we used both molecular dynamics (MD) simulation and experimental methods to explore the influence of salt ions on the electroporation process. Giant unilamellar vesicles (GUVs) were constructed as the model, and sodium chloride (NaCl) was selected as the representative salt ion in this study. The results show that the electroporation process follows lag-burst kinetics, where the lag period first appears after applying the electric field, followed by a rapid pore expansion. For the first time, we find that the salt ion plays opposite roles in different stages of the electroporation process. The accumulation of salt ions near the membrane surface provides an extra potential to promote the pore initiation, while the charge screening effect of the ions within the pore increases the line tension of the pore to induce the instability of the pore and lead to the closure. The GUV electroporation experiments obtain qualitatively consistent results with MD simulations. This work can provide guidance for the selection of parameters for cell electroporation process.

## I. INTRODUCTION

Cell electroporation is an attractive cell engineering technique, showing great potential for various biological and biomedical applications, such as genes transfection,^1^ design of permeable species,^2,3^ as well as the development of antibiotics^4^ and anti-tumor drugs.^5,6^ It is a noninvasive technique that can improve the permeability of the biological cell membrane temporarily by applying an appropriate pulsed electric field to allow foreign species to enter the interior of the cell.^7^ It has been shown that the cell electroporation process is affected by various factors, such as electric parameters, cell types, and the medium properties.^8–10^ In particular, the medium properties have been reported to significantly affect the electroporation process.^11,12^ Although a lot of efforts have been made to study the effect of ions in media on the electroporation process, the underlying mechanism is still unclear.^13–18^ For example, Ruzgys *et al.*^14^ evaluated the effect of medium conductivity on cell transfer by counting the percentage of cells killed during the electroporation-mediated transfer of bleomycin, and the result showed that the transfer efficiency is inversely proportional to the extracellular conductivity of the medium. Karal *et al.*^19^ prepared giant unilamellar vesicles (GUVs) as a simplified cellular model to investigate the effect of salt concentration on cell electroporation and discovered that the probability of ruptured GUVs in 60 s induced by electroporation increased with the decrease in salt concentration. They attribute it to the salt ion induced surface charge shielding that slow down the pore formation rate. However, Rols and Teissie^17^ found that the molecular transfer efficiency is proportional to the ionic strength of the medium by calculating the percentage of cells permeated by trypan blue after electroporation. Due to the limitations of the observation methods, the changes in membrane microstructure during the electroporation process cannot be directly recorded, leading to the controversial conclusions. Therefore, it is very significant to establish a more reliable model of the membrane electroporation mechanism to guide the cell electroporation operations.

Molecular dynamics (MD) simulations have been shown to be a powerful tool for the study of interactions between molecules within biological membranes at the atomic scale.^20–23^ Using MD approaches, a number of factors affecting the membrane electroporation process have been investigated, such as external electric fields,^20^ cholesterol,^24,25^ phospholipid type,^22,26^ and the ion concentration.^27,28^ However, due to the different parameters used in different experimental conditions, it is hard to verify the MD simulation models using the experimental results in existing literature.

In this study, we adopted both the MD simulation and the experimental method to investigate the influence of the salt ions on the electroporation process. Sodium chloride was selected as the representative salt ion, and a GUV model was constructed in the MD simulation to study the effects of the salt ions on the electroporation process. The MD results reveal that the electroporation process follows the lag-burst kinetics, where a lag period first appears after the applied electric field, followed by a rapid pore expansion. The self-resealing behavior was observed at high salt concentration. We found that the salt ion played opposite roles in the different stages of the electroporation process. At the same time, GUVs were prepared by electroformation method to perform the electroporation experimentally to verify the MD results.

## II. RESULTS AND DISCUSSION

### A. Threshold electric field of electroporation

It has been shown that the minimum electric field required for the electroporation of GUVs are closely related to the composition and concentration of the electrolyte.^29^ Therefore, we first studied the influence of NaCl concentration on the minimum electric field required for the perforation. In MD simulations, the minimum electroporation electric field for each simulated system was determined by increasing the value of the external electric field in steps of 25 kV/cm until a pore is formed within 10 ns. In the experimental study, the minimum electric field was determined as the value under which a significant deformation of the GUV and leakage of fluorescent dye were observed in 1 min.

**FIG. 1.**
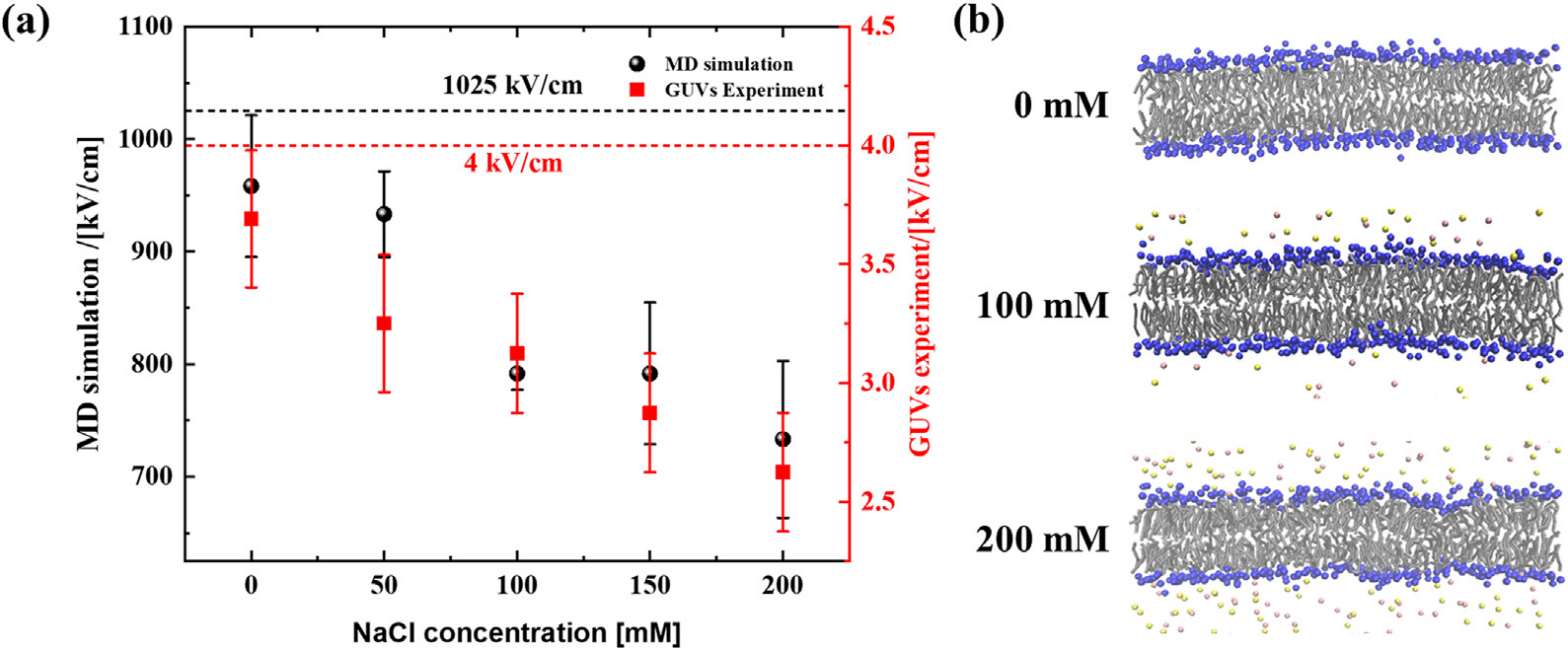
Electroporation threshold electric field: (a) the minimum electric field required for electroporation of GUVs at different NaCl concentration and (b) the schematic diagrams of the accumulation of salt ions near the membrane surface in different NaCl concentrations under the threshold electric field.

As shown in Fig. 1(a), the minimum electric field in both simulation and experiment systems decreases with an increase in NaCl concentration, indicating that cells are more prone to be perforated at higher salt concentration. However, for a given NaCl concentration, the minimum electric field between the simulation systems and the experimental systems shows large differences. The MD simulation is usually performed on the nanosecond timescale, and stronger electric field is required to realize the perforation than that performed on microsecond timescale of the experimental study. It has been reported that the lipid bilayer can be regarded as a capacitor, and the accumulation of salt ions near the membrane surface under the action of electric field would provide an extra potential of the capacitor; thus, lower electric field is required for the electroporation of membrane in the higher salt concentration solution.^30^ As shown in Fig. 1(b), more salt ions would accumulate near the membrane surface under the threshold electric field at higher NaCl concentration. We further measured the electric potential difference across the membrane for different NaCl concentrations and found that the electric potential difference was almost the same for all the NaCl concentrations with different applied threshold electric fields [Fig. S2(a)]. Therefore, we can conclude that the accumulation of the salt ions near the membrane surface can provide extra local electric field to promote the pore initiation. It is noteworthy that the accumulation of ions near the membrane would also happen without the applied external electric field, where the sodium ions would penetrate into the flexible headgroup layer to electrostatically interact with the negatively charged phosphate groups to form an electric double layer [Figs. S2(b) and S2(c)].

To ensure that electroporation of GUVs would occur in all simulation or experiment systems and minimize the influence of the applied electric field on the electroporation process, threshold electric fields of 1025 and 4 kV/cm were selected to perform the electroporation for the simulation and experiment systems, respectively.

### B. Transmembrane substance flux

**FIG. 2.**
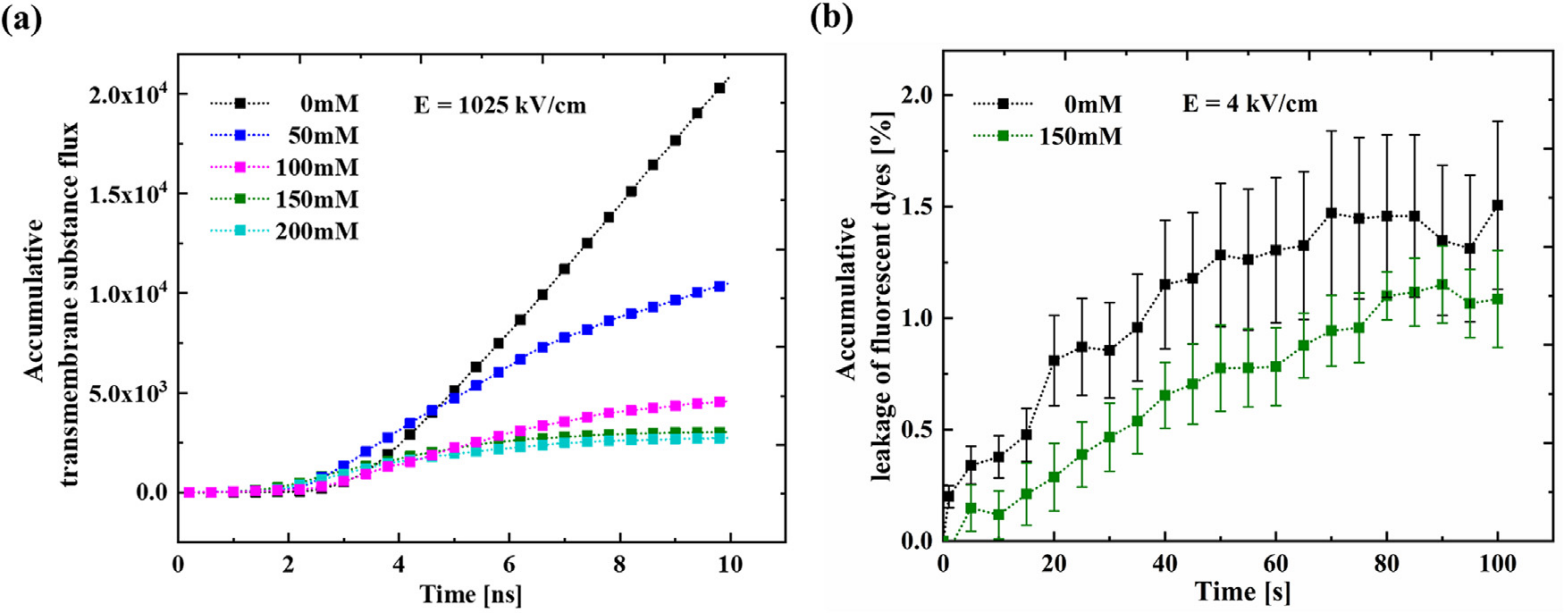
Accumulated transmembrane substance transport by electroporation: (a) the accumulative transmembrane substance flux at different NaCl concentrations using the MD method after applying an electric field of 1025 kV/cm and (b) the fluorescence dye's leakage with time at different salt concentrations using the experimental method after applying an electric field of 4 kV/cm.

In MD simulations, the accumulative transmembrane substance flux is defined as the cumulative flux of water molecules and ions from the inside to the outside of the membrane.^28^ By observing its changes over time, the efficiency of substance transfer during electroporation can be analyzed in detail. MD simulations (*E* = 1025 kV/cm) were performed in buffer solutions containing different NaCl concentrations for 10 ns to measure the accumulative transmembrane substance flux. Figure 2(a) shows the accumulative transmembrane substance flux as a function of time in the medium containing different concentrations of NaCl. It can be found that the transportation process starts after a lag period once the electric field is applied. After the lag period, the accumulative transmembrane substance flux shows a rapid increase with time for 0 mM NaCl, while it shows a gradual increase in the presence of NaCl. When the NaCl concentration is higher than 150 mM, the accumulative transmembrane substance flux reaches a plateau at the end of the process. The accumulative transmembrane substance flux at the end of the 10 ns simulation shows a decrease with an increase in NaCl concentration.

Subsequently, we performed the electroporation experiment of GUVs by loading 100 electrical pulses at an electric field of 4 kV/cm and monitored the leakage of fluorescent dye from the GUVs. The degree of the fluorescent dye leakage is characterized by the ratio of the fluorescence intensity difference before and after GUV perforation to the fluorescence intensity before perforation. Figure 2(b) shows the ratio of the fluorescent dye leakage as a function of time in 0 and 150 mM NaCl aqueous solutions. It can be found that the fluorescent dye leaked from the GUV in 150 mM NaCl aqueous solution is smaller than that in 0 mM NaCl aqueous solution during the monitoring period, which is consistent with the simulation results, indicating that the salt ion plays a role in regulating the electroporation process. However, no lag period was observed in the experimental studies.

Considering the scale difference between the MD simulation and experiment, we also simulated the cumulative transmembrane substance flux and fluorescent dye leakage concentration after electroporation of cells of 20 *μ*m diameter at 4 kV/cm for one pulse time (100 *μ*s) using COMSOL Multiphysics software (COMSOL simulation model is shown in the supplementary material). The results showed that the accumulative flux of transmembrane substance across the cell membrane increased with time and decreased with the NaCl concentration at the end of one pulse, which shows a similar trend as in the MD simulation [Fig. S4(a)]. The decreased percentage of the fluorescent dye concentration in the cell in 150 mM NaCl was lower than that in 0 mM salt concentration after one electrical pulse, which is consistent with the GUV experimental results [Fig. S4(b)]. The lag period is also less pronounced in COMSOL simulation than in the experimental study.

Because MD simulations can provide intrinsic structure information of membranes at a nanosecond timescale, more elaborate details about the perforation process can be extracted from this method than using the experimental method. In order to investigate the mechanism of salt ions affecting substance transfer efficiency, we used MD simulations in the subsequent section to investigate the electroporation process in detail at the nanosecond timescale.

### C. Kinetics of pore volume during electroporation

It has been shown that changes of the pore structure on the biomembrane play an important role in dictating the substance transportation efficiency during electroporation process,^28,31^ which is commonly measured by the change in pore cross-sectional area.^32^ However, the varied pore shape and curl degree of the membrane can lead to large variations in the cross section area measurement, thus leading to significant deviations in the resultant substance transfer efficiency.^33,34^ Therefore, this study employs pore volume to characterize the change in pore structure during formation and expansion. After perforation, the membrane pores will be filled with water molecules and salt ions, which are represented as spheres of the same diameter and are closely packed. Therefore, the pore volume can be calculated from the total volume of water molecules and ions inside the membrane pore, denoted by the following formula:

Vpore=(4/3πRm3Nions)/η, (1)

where $N_{\mathrm{ions}}$ is the total number of water molecules, sodium and chloride ions within the membrane pores, *R_m_* (=0.711 nm) is the radius of the coarse-grained particles in the simulation system,^28^ and *η* (= 0.64) is the close packing density of uniform spheres.^35^

**FIG. 3.**
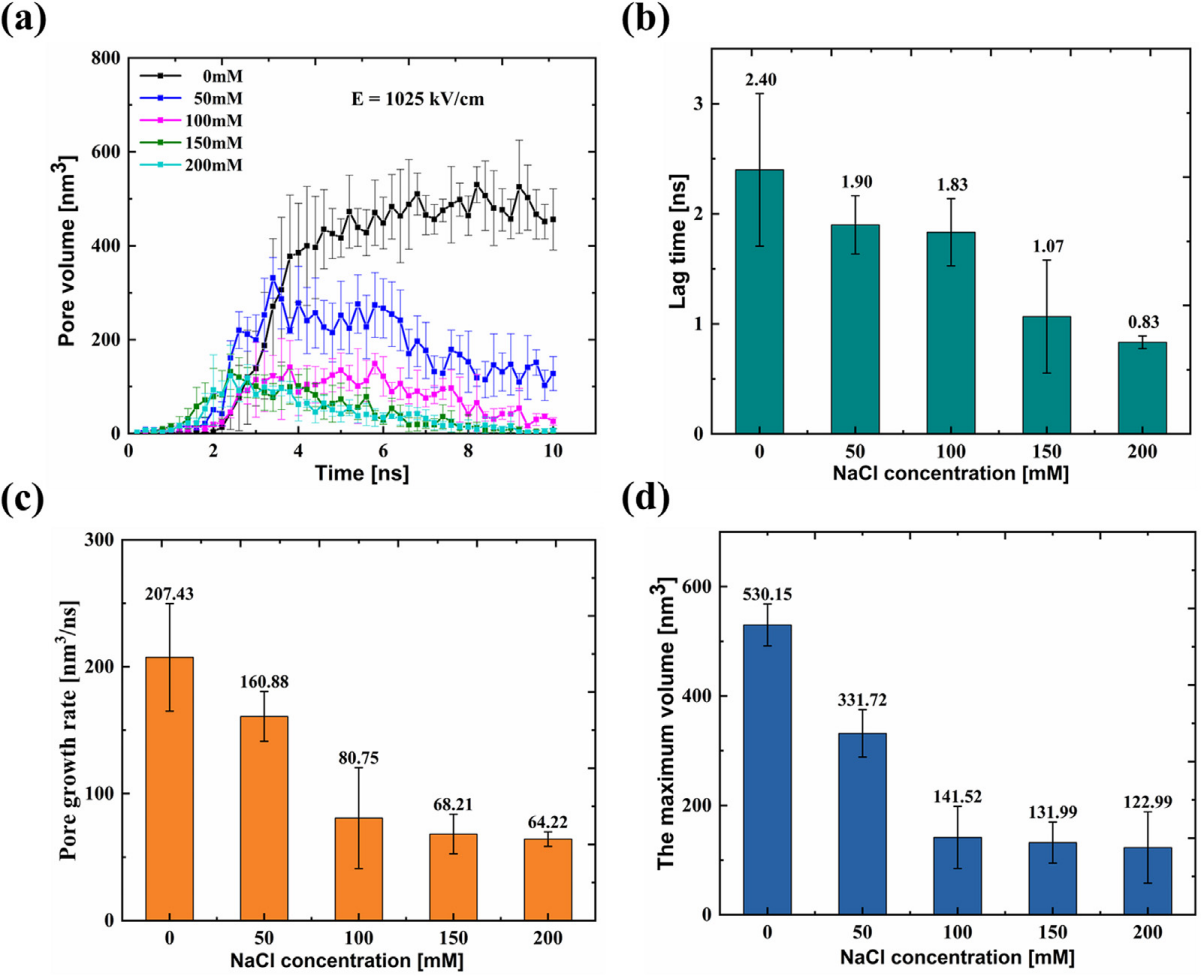
Volume change of membrane pores during electroporation. (a) Pore volume formed within the membrane after the electric field over time at different salt concentrations with the applied electric field of 1025 kV/cm. The growth curve of pore volume under different NaCl concentrations follows lag-burst kinetics: a slow growth phase (termed as lag phase, lasts ∼ 2 ns) followed by a rapid growth phase (termed as burst phase). The lag time (b), pore growth rate (c), and the maximum volume (d) of the electroporation process measured from Fig. 3(a) as a function of NaCl concentration.

Figure 3(a) shows the dynamic pore volume change with time at different NaCl concentrations. After the application of the electric field, the pore volume of the membrane in different NaCl concentrations rarely changes for a period of time (termed as lag phase), followed by an obvious increase (termed as burst phase). When there are no salt ions in the solution, after the lag period, the pore volume shows a rapid increase and finally reaches an equilibrium state. However, when ions are present in the solution, after the lag period, the pore volume first shows an increase and reaches the maximum and finally shows a gradual decrease. Moreover, with increase in the NaCl concentration, the pore volume decreases more sharply.

#### 1. Pore initiation

The lag time is the time experienced in the lag phase, that is, the time required for the pore volume to increase significantly [with a large change slope in Fig. 3(a)] from close to zero. As shown in Fig. 3(b), the lag time decreases from 2.40 ± 0.69 to 0.83 ± 0.06 ns when the NaCl concentration increases from 0 to 200 mM. It has been reported that the realignment of the dipoles of water molecules in the vicinity of the lipid bilayer induced by the local electric field induces the formation of water fingers, which initiates the pore formation.^36^ In the NaCl aqueous solution, we found that ions would accumulate in the vicinity of the polar lipid molecules (Fig. S5), providing an additional local electric field to facilitate the pore initiation. More salt ions in the higher salt concentration solution would accumulate near the membrane surface and provide a higher extra potential to promote the pore initiation, leading to a shorter lag time required for the pore formation.

#### 2. Pore growth

The slope of the plot of the burst phase shows the pore growth rate of the membrane under the electric field. Once hydrophilic pores were formed, they expanded rapidly in the radial direction. The rate of pore growth decreases from 207.43 ± 42.34 to 64.22 ± 5.65 nm^3^/ns as the NaCl concentration increases from 0 to 200 mM [Fig. 3(c)]. The maximum volume of the pore achieved during the electroporation process also shows a decrease with the increase in the NaCl concentration [Fig. 3(d)]. The addition of the salt ions would interact with the polar phospholipid molecules to lead to the tight arrangement of lipid molecules, which makes it hard for pore expansion. We have calculated the surface area of membrane and found that the width of the membrane decreased from 20.461 to 20.376 nm when the NaCl concentration increased from 0 to 200 mM [Fig. S6(a)]. The resultant area per lipid molecule shows a decrease from 61.20 ± 0.09 Å^2^ for 0 mM to 60.85 ± 0.07 Å^2^ for 200 mM [Fig. S6(b)]. Therefore, the higher NaCl concentration leads to a smaller pore growth rate and a maximum volume of the pore.

#### 3. Pore resealing

**FIG. 4.**
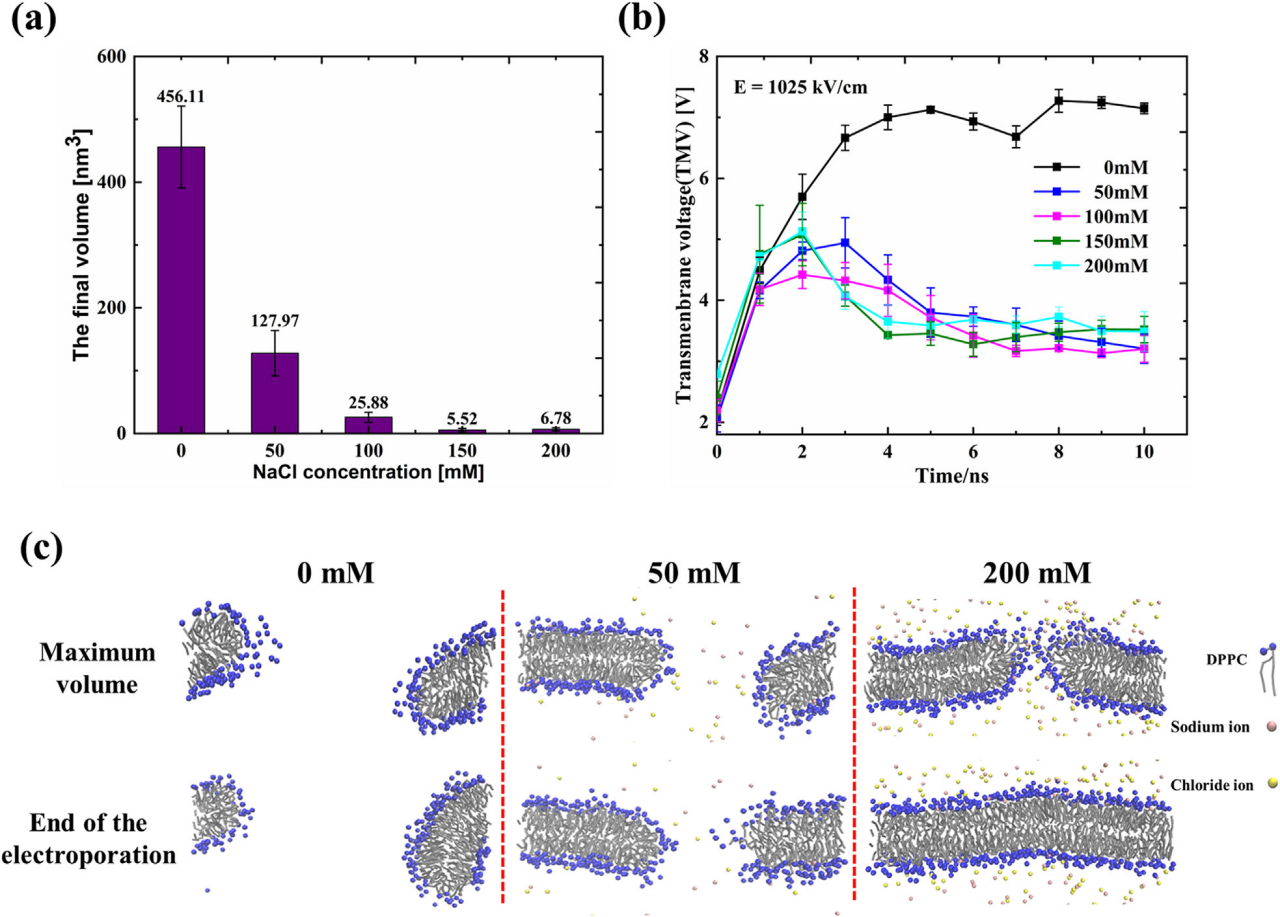
Self-resealing behavior of electroporated pores at 1025 kV/cm electric field: (a) the final volume of pores at 10 ns for different NaCl concentrations, (b) the change of transmembrane voltage with time at different NaCl concentrations during electroporation process, and (c) schematic diagrams of pore structure for different concentrations of NaCl at maximum volume and at the end of the electroporation (at 10 ns).

After the burst phase, the pore volume of the membrane in 0 mM NaCl solution was kept stable with the final value of 456.11 ± 65.14 nm^3^ [Fig. 4(a)]. However, the final volume of pores in NaCl solution was much smaller than that at the end of the burst phase. In particular, the final volume of pores was 6.78 ± 2.45 nm^3^ with the NaCl concentration of 200 mM [Fig. 4(a)], showing a self-resealing behavior of the pore. It has been reported that there exist two terms to determine the stability of the pores: the lateral electrical tension and the line tension, respectively.^37^ The transmembrane voltage (TMV) induced lateral electrical tension favors the pore expansion, while the line tension prefers to close the pore. The arrangement of the lipid molecules on the pore edge is responsible for the line tension.^38^

Previous studies have suggested that the TMV is the main reason to determine the stability of the pore after the pore formation.^39–42^ After the formation of pores, water molecules and ions flow through the pores and form the transmembrane current, leading to a decrease in TMV, and thus resulting in the reduction in pore volume and even the self-resealing behavior of pores.^43^ We measured the TMV variations with time at different NaCl concentrations after applying the electric field of 1025 kV/cm [Fig. 4(b)]. It is found that the TMV rises rapidly to a maximum value and remains stable for 0 mM NaCl, while the TMV first increases and reaches a maximum and then shows a slight decrease in the presence of NaCl. However, the final TMVs are almost the same for all NaCl concentrations in the range of 50–200 mM, indicating the decrease in TMV is not the main cause for the resealing behavior of pores.

The electrostatic repulsion between the polar lipid molecules is important to maintain the stability of the hydrophilic pores. It has been shown that the interaction of salt ions with the polar lipid molecules on the pore line would increase the line tension by stiffening the membrane and thus lead to the destabilization of the pore.^44^ We further investigated the pore structure change after reaching the maximum pore volume and found that more salt ions would interact with the lipid molecules within the pore at higher NaCl concentrations [Fig. 4(c)]. Therefore, it is reasonable to conclude that after the pore formation, ions flow through the pores and interact with the polar lipid molecules on the pore edge, causing the instability of the hydrophilic pores and, thus, leading to contraction of pores and even the pore closure. The pore initiation on the membrane is a fast process, while the rearrangement of phospholipid molecules for the pore expansion is slow.^45^ Thus, the pore on the membrane is first formed and expanded after the lag period, and then gradually shows closure.

Our simulation results show that the salt ion plays opposite roles in the different stages of the perforation process. In the lag phase, ions would accumulate near the membrane surface, providing an additional potential to increase the lateral electrical tension to promote the pore initiation. After the pore formation, the charge screening effect induced by the salt ions contributes to line tension increase in the pore, leading to the slow pore growth rate and the closure of the pore.

### D. Application and validation

When using electroporation to transport exogenous substances (such as DNA, RNA, proteins, and drugs) into the cell, the efficiency of the transport has been the focus of the research.^10,12,14^ Based on our results, in order to improve the transfer efficiency, it is possible to reduce the ion concentration once the pore is formed to slow down the closure rate of the pore. However, this protocol is difficult to be realized in practice because the duration of the entire electroporation process is too short and there is not enough time to change the ion concentration of the buffer solution once the pore forms. As an alternative, increasing the electric field strength to increase the lateral electrical tension to stabilize the pore is also an option.

**FIG. 5.**
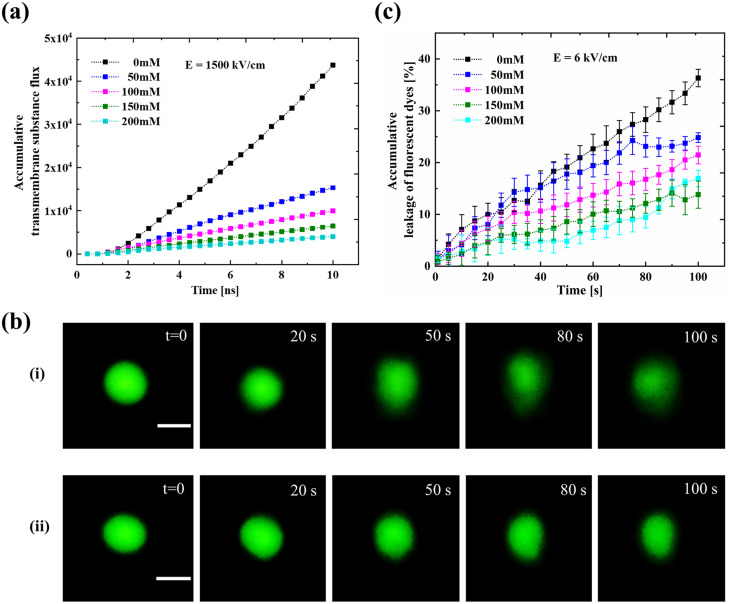
Accumulated transmembrane substance transport during electroporation: (a) the transmembrane substance flux after applying the electric field of 1500 kV/cm at different NaCl concentrations, (b) time-sequential fluorescence images of electroporated GUVs at 6 kV/cm in (i) 0 mM and (ii) 150 mM NaCl solutions (scale bar: 20 *μ*m), and (c) the variation of the fluorescent dye leakage with pulse time at different salt concentrations at 6 kV/cm.

In order to verify this strategy, both the simulation and experimental studies were conducted. In order to ensure that all the formed pores can be maintained for more than 5 ns, the applied electric field of 1500 kV/cm was selected in the MD simulation, which is higher than the minimum stable electric field strength (MSEFS) for all NaCl concentrations [Fig. S7(a)]. The dynamic pore volumes in all NaCl concentrations reach a stable state at the end of the 10 ns simulation without showing the closure behavior [Fig. S7(b)]. Figure 5(a) shows that the accumulative transmembrane substance flux increases continuously with time after the lag period after applying the electric field of 1500 kV/cm, and no plateau was found at higher NaCl concentrations. The electric field of 1500 kV/cm was selected in the simulation study, which is approximately 1.5 times the simulated threshold electric field (1025 kV/cm). Thus, the corresponding experimental electric field was chosen at 6 kV/cm, which is also 1.5 times larger than the experimental threshold electric field (4 kV/cm). After applying the electric field of 6 kV/cm, the GUVs show an obvious deformation and the fluorescence intensity of the GUVs decreases with time [Fig. 5(b)]. The accumulative leakage of the fluorescent dye in GUVs shows a similar trend as in the simulation results, showing an increase with time [Fig. 5(c)]. Therefore, selection of appropriate electric field strength is helpful to maintain the membrane pore to increase the transfer efficiency.

**FIG. 6.**
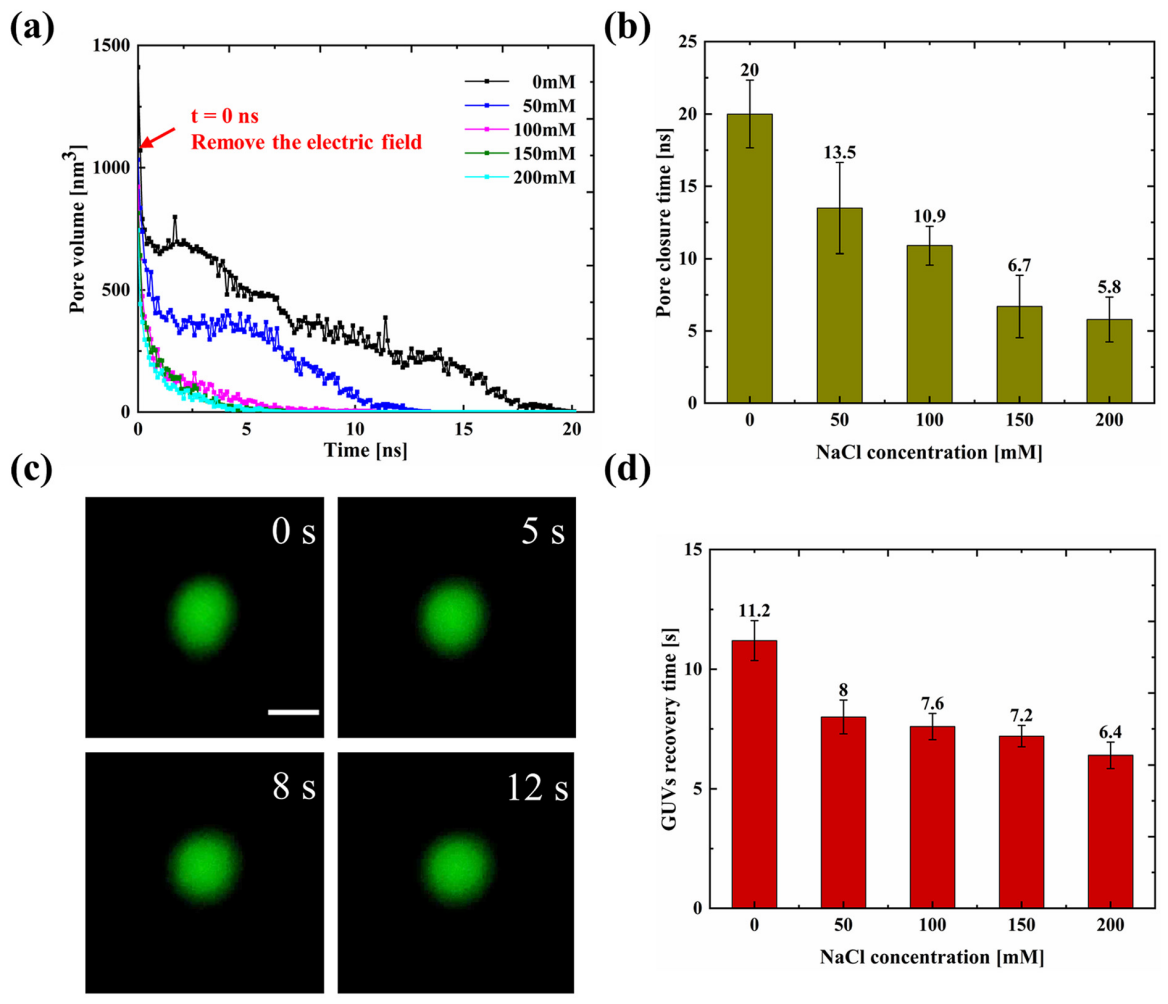
Pore closure process after removal of applied electric field: (a) the pore volume changes with time, (b) the time required for pore closure after removing the external electric field of 1500 kV/cm for different NaCl concentrations, (c) time-sequential fluorescence images of GUVs in 0 mM NaCl solutions after removing the applied electric field of 6 kV/cm (scale bar is 20 *μ*m), and (d) the recovery time for GUVs to return to a spherical shape after removing the applied electric field of 6 kV/cm under different NaCl concentrations.

We further removed the applied electric field of 1500 kV/cm in MD simulation and found that the pore volume showed a decrease with time for the NaCl concentration in the range of 0–200 mM [Fig. 6(a)]. The time required to completely close the pores of the membrane decreases from 20 ± 2.33 to 5.8 ± 1.56 ns with the NaCl concentration increasing from 0 to 200 mM [Fig. 6(b)], which further confirms that salt ions would promote the contraction of phospholipid membrane. Figure 6(c) shows that the shape of GUV quickly returns to the spherical shape after removing the electric field of 6 kV/cm, which can indirectly reflect the progress of pore resealing. The time required for the GUVs to return to the spherical shape decreases from 11.2 ± 0.84 to 6.4 ± 0.55 s when the NaCl concentration increases from 0 to 200 mM [Fig. 6(d)], showing a similar trend as in the simulation results.

## III. CONCLUSIONS

In summary, MD simulation and experimental methods were conducted to investigate the influence of salt ions on the electroporation process of GUVs. The MD simulation can provide more elaborate details about the electroporation process than experimental methods. The experimental phenomena based on GUV perforation are qualitatively consistent with the MD simulation results in most aspects. The pore formation during the electroporation process was found to follow a lag-burst kinetics. For the first time, we found that the salt ion plays opposite roles in different stages of the electroporation process. The existence of salt ions would promote the pore initiation, while they would prevent the further expansion of the pore and can further lead to the pore closure after the pore formation, which is not completely the same as the traditional cell membrane electroporation theory.

In the lag period, the accumulation of salt ions near the membrane surface would provide an extra local electric field to increase the lateral electrical tension to promote the reorganization of water and lipid molecules and, thus, promote the pore initiation. After the pore formation, interaction of the salt ions with the polar lipid molecules induced close arrangement of lipid molecules, which makes it hard for the reorientation of lipid molecules, leading to the slow pore growth rate. The salt ions move across the membrane and interact with the polar lipid molecules on the pore edge, which further increases the line tension, leading to the instability of the pores and even the pore closure. This process is relatively slow, which has a great impact on the stability of membrane pores in the middle and late stages of perforation. This work theoretically explains the effect of ion concentration on the microscopic mechanism of cellular electroporation and can provide guidance for the selection of cellular electroporation buffers.

## IV. METHODS

### A. Materials

Egg phospholipid (L-α-phosphatidylcholine, PC, 90%) was purchased from Aiweituo (Shanghai, China). Chloroform was bought from Chuandong Huagong (Chongqing, China). Fluorescent dye, 3′,6′-dihydroxy-3-oxospiro[isobenzofuran-1(3H) (CF, Green), was bought from Psaitong (Beijing, China). Sodium chloride was purchased from Dongfang Huabo (Chongqing, China). Polydimethylsiloxane (PDMS) and polymethyl methacrylate (PMMA) for microchip fabrication were purchased from Dow Corning (Midland, USA) and Yikang (Shanghai, China), respectively. Milli-Q water with a resistance of 18.2 MΩ·cm was used for aqueous solution preparation.

### B. MD simulations

The MD simulations employed in this study were based on a coarse-grained molecular dynamics (CG-MD) method,^46^ and the interactions between coarse-grained sites were modeled using the MARTINI force field.^47^ All simulations were performed using the GROMACS 2020.2 package.^48^ In addition, the Visual Molecular Dynamics (VMD) 1.9.2 package^49^ was used to visualize the simulation results.

**FIG. 7.**
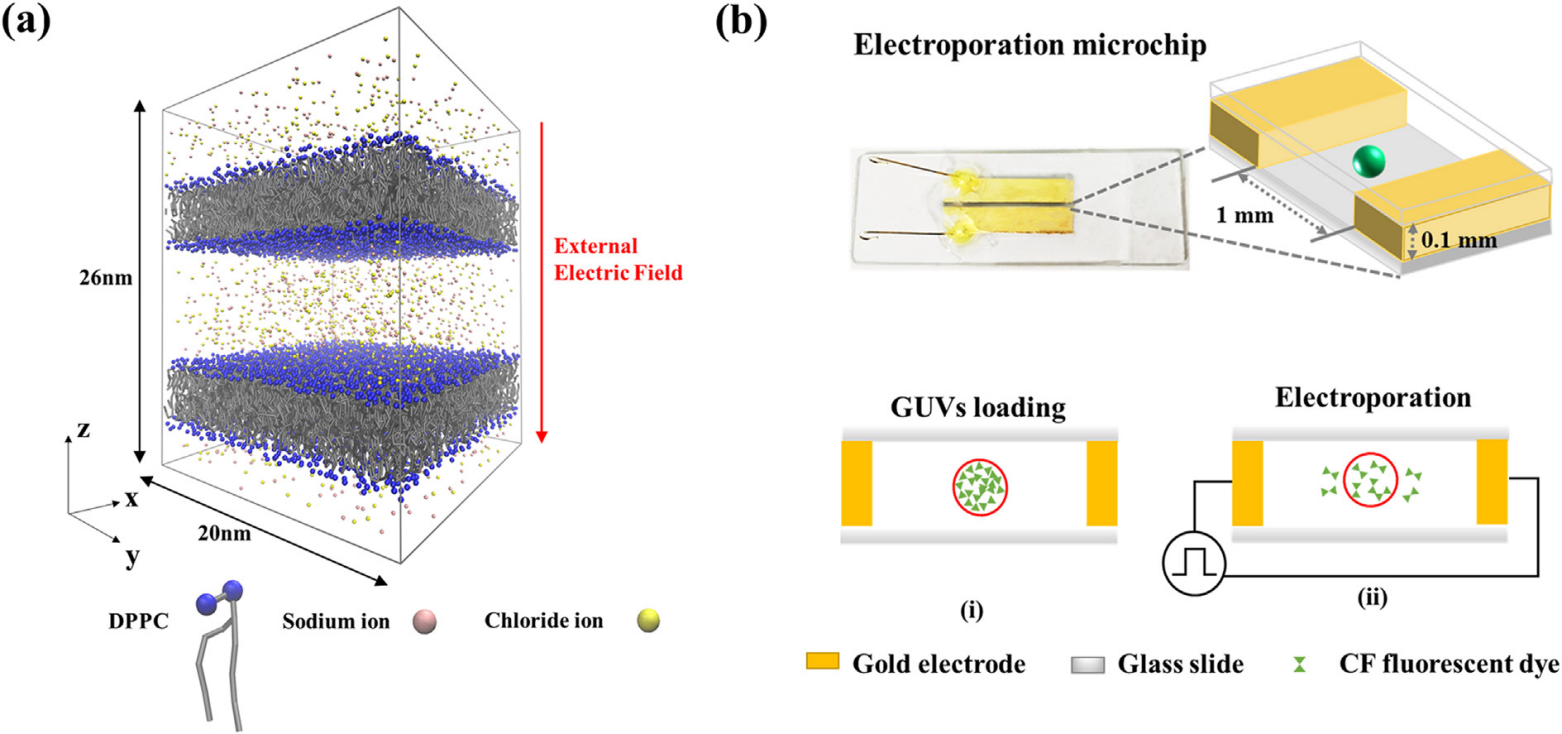
Simulation model and experimental microchip. (a) Schematic diagram of the MD simulation system in 150 mM NaCl solution. (b) Design of the microchip and experimental procedures for GUV electroporation.

The size of the simulation system was 20 × 20 × 26 nm^3^, including two phospholipid double-layer membranes and two solution chambers as shown in Fig. 7(a). The phospholipid membrane with such a small dimension can be regarded as flat, and the influence of the curvature would not be discussed in this study. The phospholipid membrane is composed of 1,2-dipalmitoylphosphatidylcholine (DPPC). The chamber solution consisted of sodium ions, chloride ions, and coarse-grained water molecules. The water molecules were simulated using the polarized water model.^47^ NaCl concentrations varied from 0 to 200 mM, which covered the concentration of the isotonic saline solution.^32^ The NaCl concentration is kept equal in the outer and inner solvent chambers to eliminate the influence of ionic imbalance and osmotic pressure on the electroporation results. Table S1 shows the detailed compositions of the entire system.

The MD simulations were performed under the conditions of NPT (constant pressure and constant temperature) along the membrane normal. The temperature and pressure were maintained at 323 K and 1 bar using the velocity-rescaling method with a time constant of 1 ps and the Parrinello–Rahman pressure coupling method with a time constant of 12 ps, respectively. Lennard-Jones potentials for van der Waals interactions were calculated within a cutoff length of 1.2 nm, and the particle mesh Ewald (PME) method was used to calculate the electrostatic interactions. Periodic boundary conditions were applied in all directions. Finally, the electric field was applied downward perpendicular to the membrane surface [Fig. 7(a)]. The simulations have been performed 4–8 times for each system under the same conditions to ensure data reproducibility.

It should be noted that the lower lipid bilayer membrane is constrained to a post-equilibrium state and remains unchanged. The reason for this is to prevent the concomitant electroporation behavior of the upper lipid bilayer,^32^ since the distance between the two bilayers (∼10 nm) in our simulation model is much smaller than the typical cell size.

### D. Preparation and electroporation of GUVs

#### 1. Preparation of GUVs

Electroformation method was adopted to prepare GUVs.^50^ The microchamber used for GUV preparation is a sandwich microstructure consisting of two indium tin oxide (ITO) glass electrode substrates spaced by a PDMS layer (Fig. S1). Typically, 60 *μ*l of chloroform solution containing 2 mg/ml phospholipid was dropped onto the bottom substrate of the microchamber to allow it to naturally spread, followed by degassing in a vacuum oven for 30 min to completely evaporate the solvent to form a lipid film. Then, 1 ml aqueous solution containing 10 *μ*M fluorescent dye, carboxyfluorescein (CF), and specific concentration of NaCl was added slowly into the chamber, followed by applying a sinusoidal voltage (3 Vp-p, 10 Hz) for 30 min. The resultant GUVs were extracted by centrifugation at 500 rpm for 3 min and then re-dispersed in a NaCl solution with the same concentration as in the interior of GUVs.

#### 2. Electroporation of GUVs and measurements

The microchip used for electroporation contains a pair of coplanar gold foil microelectrodes fixed on the bottom glass substrate with PDMS as the adhesive [Fig. 7(b)]. The size of each microelectrode is 25 mm (L) × 5 mm (W) × 0.1 mm (H), and the two electrodes were aligned with a gap of 1 mm. For GUV electroporation, 20 *μ*l of GUV suspension was first added to the gap between the two microelectrodes, and a glass slide was then covered on top of the gap [Fig. 7(b)]. A pulse signal (amplitude: 200–600 V, duration: 100 *μ*s, interval between two pulses: 1 s, pulse number: 100) was then applied to the electrodes to perform the electroporation using an electroporation generator (BEX LF301, Japan).

Fluorescence microscope (Olympus BX61, Japan) equipped with a CCD camera (Mshot MXS2, China) was used to monitor the GUV electroporation process by recording the leakage of the fluorescent dye from the GUVs. The fluorescence images were acquired by the CCD camera with a 5 s acquisition interval, and the fluorescence intensities were measured using ImageJ software (National Institutes of Health, USA).

## SUPPLEMENTARY MATERIAL

See the supplementary material for the details of additional simulation data and experimental procedures.

## Data Availability

The data that support the findings of this study are available from the corresponding authors upon reasonable request.
